# The “Clinician’s illusion” and the epidemiology, diagnosis and treatment of depressive disorders

**DOI:** 10.1186/s12888-018-1969-3

**Published:** 2018-12-20

**Authors:** Scott B. Patten

**Affiliations:** Department of Community Health Sciences and Department of Psychiatry, University of Calgary. 3rd Floor TRW Building, 3280 Hospital Drive NW, Mathison Centre for Mental Health Research & Education, Hotchkiss Brain Institute and the Alberta Children’s Hospital Research Institute, Calgary, AB T2N 4Z6 Canada

**Keywords:** Simulation studies, Major depressive disorder, Depressive disorders, Mathematical models

## Abstract

**Background:**

Depression often occurs in association with stressful events. However, people with depressive disorders may experience episodes in response to minor stressors or “out of the blue.” Similar episodes can occur in people who do not have a disorder in response to severe events. This plurality of symptom patterns, occurring as it does in the absence of precise demarcation from normality has led to controversy over how depressive disorders should be defined, how common they are, and when treatment should be offered. Much of the controversy, however, may be illusory, arising from a tendency to view depressive disorders as defects or disease processes (the “clincian’s illusion”). Avoiding the illusion involves understanding depression as a defense rather than a defect and requires consideration of aspects of signal detection theory and the associated “smoke detector” principle. This perspective may help to understand aspects of depressive disorders that are otherwise puzzling and controversial.

**Methods:**

In this paper, implications of signal detection theory and the “smoke detector principle” are explored: (1) conceptually, (2) using calculations performed in a spreadsheet and (3) using an agent-based model. Depressive episodes are conceptualized or represented as all-or-nothing phenomena activated in response to stressful life events. These events occur in an environment that also includes variable levels of baseline stress, creating a signal detection problem. The agent-based framework allows interaction with the environment as agents attempt to achieve an ideal level of adaptation.

**Results:**

The smoke detector principle, if valid, may explain otherwise puzzling and controversial features of the depressive disorders, such as their lack of precise demarcation from normality, the role of life events and stressors and their patterns of prevalence.

**Conclusions:**

Signal detection concepts help to avoid the “clinician’s illusion” in which aspects of functioning of the body’s defenses are mistaken for a disease entity or defect. These principles emphasize inevitable difficulties that are encountered in attempts to conceptualize depressive disorders without reference to the environment in which they occur, and without addressing possible stochastic (randomly varying) elements. Because of the “clinicians illusion”, current research priorities, as well as diagnosis and treatment strategies, may be flawed.

**Electronic supplementary material:**

The online version of this article (10.1186/s12888-018-1969-3) contains supplementary material, which is available to authorized users.

## Background

Depressive disorders are often conceptualized with reference to their presumed etiology [1], for example in the past as a “chemical imbalance” [2] or more recently as a disorder of brain circuits [3]. The expectation that an unknown neurobiological defect underpins depression is an influential one that guides both clinicians and researchers. On the other hand, evolutionary models of depression focus on depression’s possible adaptive functions, which seem necessary to explain its evolutionary persistence. Durisko et al. [4], for example, categorized possible adaptive functions as those involving: energy reallocation, energy conservation, the signaling of social defeat, or soliciting resources from others. Hagen [5, 6], and subsequently Rosenstrӧm [7] developed a “bargaining model” which explains depression as a mechanism to deliver a message to a social group: that there are insufficient benefits to investing in joint enterprises. This reflects bargaining in the sense that the depressed person withholds participation until better terms are “negotiated.” Proponents of this model have argued that it may account for suicidal ideation better than other adaptive models [7] and may help to explain the consistently higher prevalence in women [8]. Another theory is that painful emotions may resemble physical pain in being a negative experience that has adaptive functions by deterring harmful actions. This possibility is called the “psychic pain hypothesis” [9].

This paper is not concerned with explanations for why depression may be adaptive in some circumstances, but rather seeks to explore some of the implications of the idea that depression can be an adaptive response. Hagen has pointed out that most evolutionary theories accept the idea that sadness, in its usual manifestations, has an adaptive purpose and that depressive disorders represent some form of dysregulated sadness [9]. This idea provides at least a common theme to various evolutionary theories, as mentioned in the preceding paragraph. It is also consistent with DSM-5’s assertion that a mental disorder must reflect a “dysfunction in the psychological, biological, or developmental processes underlying mental functioning” [10]. Wakefield’s “harmful dysfunction” model [11] also posits that depression can only be considered a clinical disorder in circumstances in which (in addition to causing harm) it represents a dysfunction of an evolutionarily designed natural function. Nesse [12, 13] grouped depression together with various other bodily mechanisms (as diverse as panic, coughing and fever) that can be regarded as defenses useful in responding to environmental threats, a concept that subsumes several types of threats and adaptive roles. When depression is described as a “chemical imbalance” or as a “disorder of brain circuits” or any of several other analogy-based disease models for depression (see [1] for other examples) a “clinician’s illusion” may be in evidence: a defense is being misinterpreted as a disease or defect. Understanding depression as a defense often seems reasonable when symptoms are mild, self-limited and occur predictably in response to life events. However, in a depressive disorder such as Major Depressive Disorder [10], symptoms may be severe, unpredictable, and maladaptive.

If depression acts as a defense, its functioning must depend on a mechanism for detection and activation in response to a threat. The problems of detection and activation mean that signal detection theory may assist with understanding aspects of this condition. The “clinician’s illusion” (which ignores signal detection) may have negative effects on research and clinical practice.

### Signal detection theory

Nesse et al. applied signal detection theory to psychiatric phenomena, invoking the so-called “smoke detector principle” [12, 13]. Nesse et al. illustrated this concept using the example of panic attacks, but posited that the same principles may apply to depression. A component of the smoke-detector concept is the idea of an all or nothing response, e.g. a panic attack, being a type of fully activated anxiety response, qualitatively different from various levels of anxiety that may be experienced in routine situations. The value of an all or nothing response system, such as the panic response, is its ability to permit a more rapid and thereby potentially more effective reaction to an environmental danger. By way of illustration, Nesse et al. considered the example of an impala that hears a sound behind a bush. The sound is too loud to be caused by a mouse and not loud enough to be definitely caused by a lion. Given adequate time, the impala might investigate the source of the sound and then decide how to respond. However, the slowness of this strategy would pose risks. Even before the situation is fully understood, the panic system provides an opportunity to prepare for danger. Nesse et al. asserted that the value of such a system can be understood in terms of its utility. The costs of a false positive response (burning an excess quantity of calories, disrupted feeding etc., in this example) are outweighed by the cost of a false negative response (which may include a risk of death or serious injury), so the optimal system, acting on incomplete information, may tolerate false positive responses in order to avoid false negative responses. Both false positive and false negative responses are associated with negative effects (disutility); but in this case, disutility is strongly weighted towards false negative responses.

This “smoke detector principle” builds on an analogy: that false alarms arising from, for example, burned toast in a toaster may be acceptable if they allow a more rapid response to a real fire. Again, the costs of a false positive response (annoyance of resetting the alarm, some wasted time) are outweighed by those of a false negative response (the house fire getting larger before it is detected).

The problem is also familiar to clinicians who engage in screening, the goal of which is earlier detection of pre-clinical disease. Since the outcome of a false negative screening test can be catastrophic, such tests must be highly sensitive, even though high sensitivity often comes at the expense of specificity and consequently a sizable number of false positive screening results. The net benefit of screening comes in a favorable trade-off between the benefits of earlier detection for some and the harms created by false positive test results (repeated tests, unnecessary time and expense, complications of biopsies etc.). The costs, benefits and harms that accompany screening procedures determine its appropriateness in various clinical contexts [14, 15]. Furthermore, the utility of screening is largely determined by its context. Screening activities that are worthwhile in high-risk populations often do more harm than good in low risk populations. The predictive value of a screening test is often poor in low base-rate situations such that harms often outweigh benefits.

In each of the situations described above, the goal is an earlier response in a situation of incomplete certainty and the best solution in terms of decision-making depends on the costs associated with signal detection errors and the gains achievable by earlier detection.

### Benefits and costs of depression

A signal detection theory of depression depends on the idea that experiencing depressive symptoms in an appropriate situation is adaptive. According to this theory, there are costs associated with becoming depressed (the costs of the defense) and costs associated with not becoming depressed in a situation in which depression would be adaptive. Disutility is conceived as the overall harm associated with both situations.

In terms of this concept of disutility, it is widely accepted that depression comes at a cost. According to the DSM-5 it is, by definition, associated with psychosocial dysfunction [10]. It is also associated with increased chronic disease incidence [16] and premature mortality [17, 18] some of which may in turn be related to activation of inflammatory processes [19]. It may also convey increased allostatic load [20]. In view of this, the optimal threshold for activation of a depressive response theoretically depends on three factors: (1) the disutility that would arise from failing to become depressed, or having a delayed onset of depression, during a time when depression would be adaptive, (2) the disutility that arises from experiencing depression, such as that due to psychosocial dysfunction, inflammation or allostatic load and (3) the frequency of situations in the environment (e.g. losses, threats and stresses) in which depression would be adaptive. The latter point is less obvious than the first two but will be familiar to those involved in screening activities, as mentioned above. The familiar “base rate” problem in screening arises because of the conditional nature of predictive probabilities. For example, the predictive value of a positive diagnostic test depends not only on its sensitivity and specificity but also on the base rate of the targeted condition. Similarly, the probability that Nesse’s antelope has heard a lion depends on whether there are any lions around, and how many.

### A static signal detection model

**Fig. 1:**
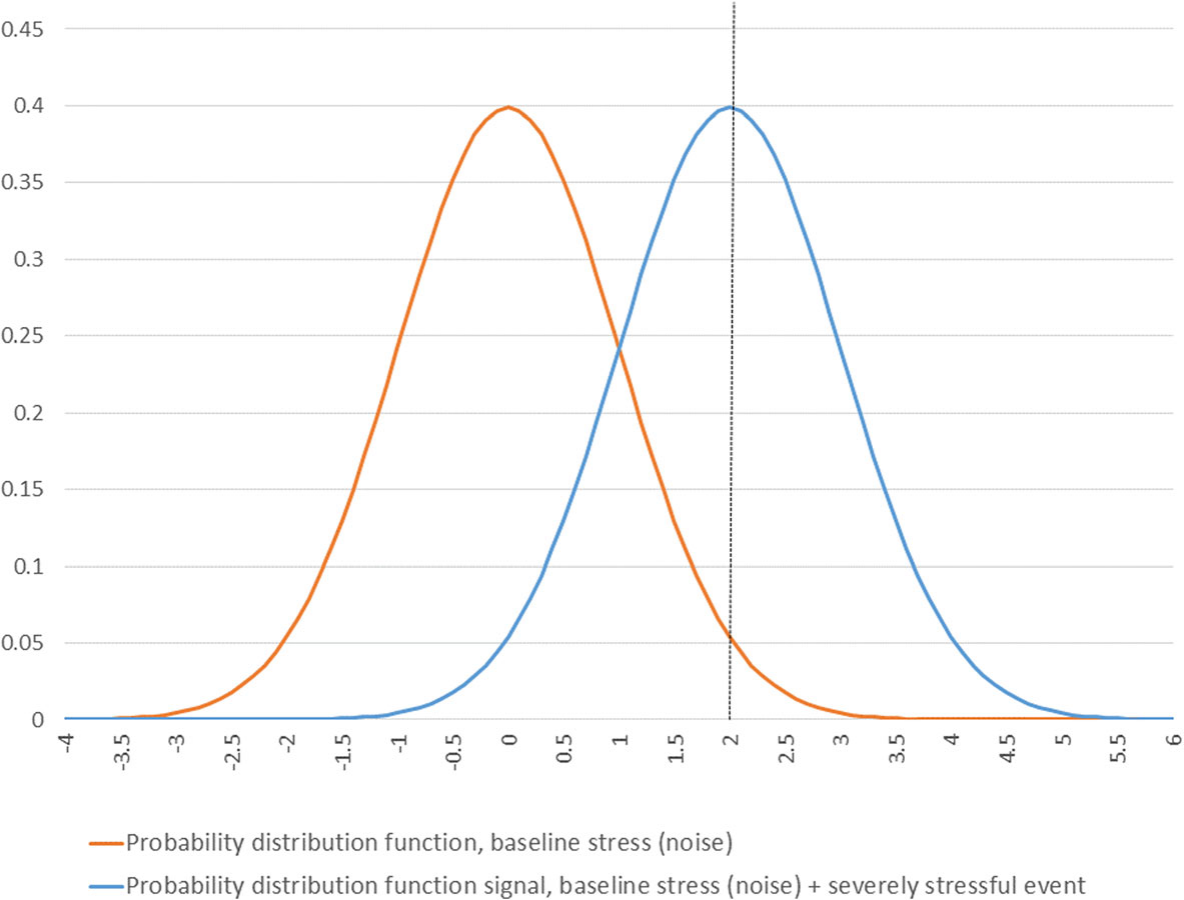
A classical signal detection paradigm

Some basic features of the signal detection concept can be illustrated using output from a static model, such as that presented in the spreadsheet attached, see Additional file 1, from which the following Figures derive. For a more detailed presentation than that presented here, see Nesse [12]. Figure 1 shows the classical signal detection scenario. The orange distribution, depicted as a standard normal distribution, represents noise in the environment, a distribution of stress that occurs in the absence of a severe event. The blue distribution represents the signal of a severe life event, offset by two units from the noise distribution. The challenge is to detect the signal despite the noise. The vertical dashed line represents an example of a signal detection threshold. An environmental reading that arises from the signal distribution and falls to the right of this line is a true positive but such a reading that arises from the noise distribution is a false positive signal. The Figure represents the probability of each type of signal through the areas under the respective curves. A false negative scenario depicted as the area under the blue curve falling to the left of the vertical line. A true negative observation is the area under the orange curve to the left of the line.

When a depressive episode is triggered by such a signal, the signal may be a true or false positive. If the signal was a true positive, then the adaptive nature of depression may eliminate some or all of the harms associated with the severely stressful event. For simplicity, in this initial example it is assumed that these costs are eliminated by activation of the depressive response. The costs in this situation are attributable to the costs of being depressed. The same cost applies to false positive activations. False negatives are associated with costs arising from the severely stressful event since this is not offset by the adaptive properties of the depressive response. A disutility function requires that costs be assigned to each contingency. The resulting disutility function depends on the costs assigned, and increases with the frequency of such events in the environment.

**Fig. 2:**
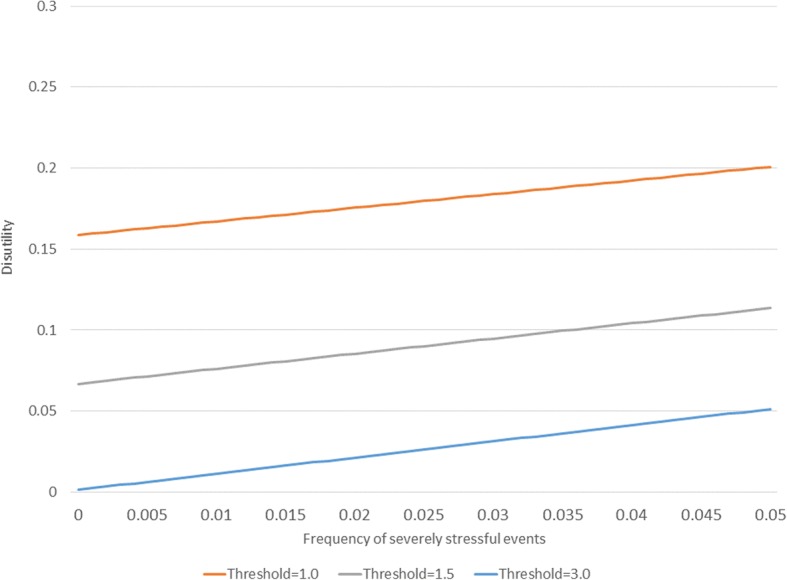
Examples of disutility as a function of the prevalence of severe events when costs of defense and harms are equal

**Fig. 3:**
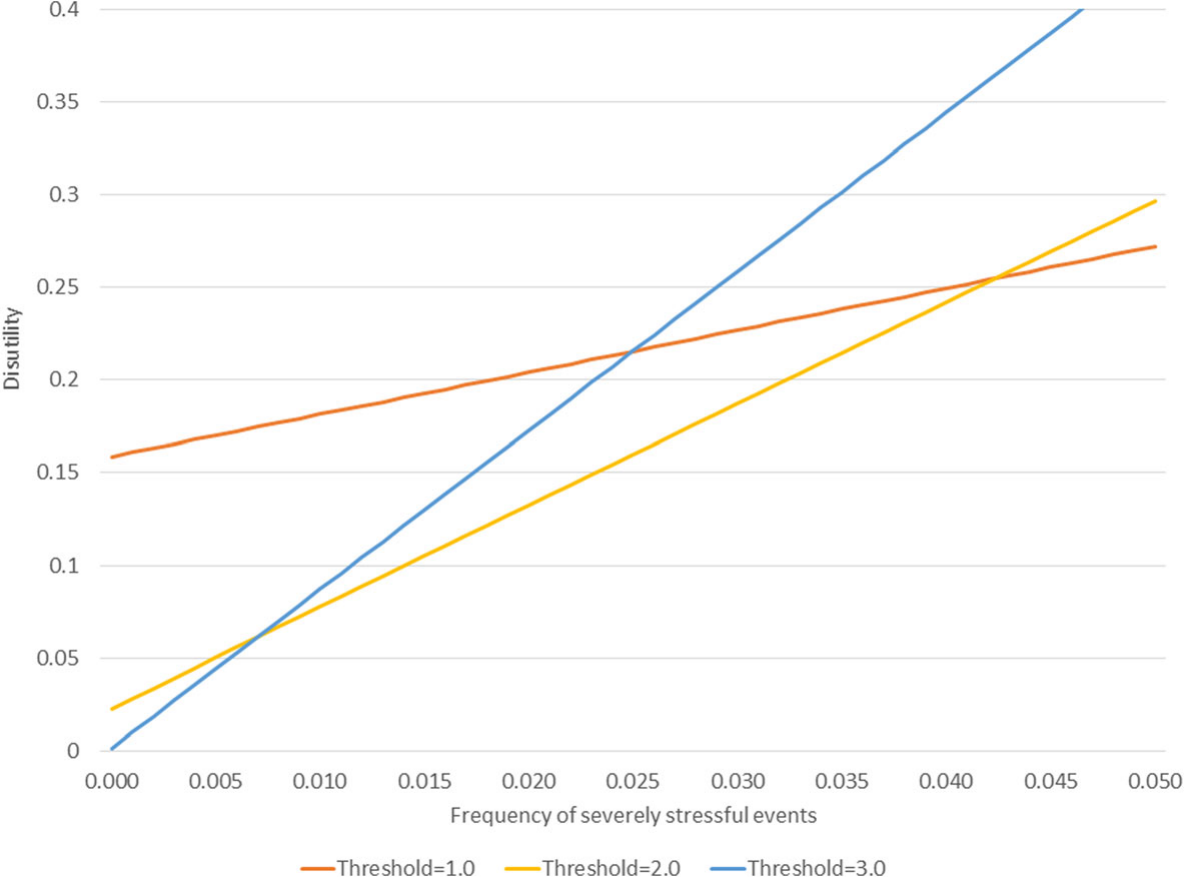
Examples of disutility as a function of the prevalence of severe events when costs of harms outweigh those of defense

Figure 2 shows the disutility function when the two costs are equal (they have both been set to a value of 1 in the underlying calculations, no units are assigned since these would be arbitrary). The Figure depicts the disutility function under three thresholds for activation of a depressive response: 1, 2 and 3. The x-axis depicts the frequency of occurrence of severely stressful events in the population. Recall that the noise distribution is a standard normal distribution, so these thresholds correspond to 1, 2 or 3 standard deviations from the mean of zero. Disutility increases with the frequency of events, irrespective of the threshold. The lower thresholds perform poorly since they have more false positive results than higher thresholds, irrespective of the frequency of harms in the environment. The higher false negative rate that occurs with higher thresholds does not change the relative ranking since the defense and the harm in this scenario have the same weight. Figure 3 depicts the same scenario, except that the cost of severely stressful events has been set to 10 while that for depression remains 1. Now the high thresholds are associated with more disutility when the frequency of events is high owing to the occurrence of false negatives. The best threshold for detecting the signal now depends on the underlying frequency of events.

Figure 2 and Figure 3 make the general point that the utility of an adaptive stress response depends on the threshold for its activation. When there is strong disutility associated with severely stressful events (as in Fig. 3), low thresholds are generally better (less disutility) when event rates are high and high thresholds are better when severely stressful event rates are low. This model is simplistic because it is static. It is likely that stress response systems can be calibrated to environmental conditions, which may introduce random variation. In order to explore the signal detection concept in a more dynamic way, an agent-based simulation model is now presented.

## Methods

A simulation model (included in the paper as offers the opportunity to represent stochastic features, which is an attractive feature for modeling a problem such as depression that often has unpredictable manifestations. NetLogo is the software used in the simulations that follow [21].

The model (included in the manuscript as Additional file 2) depicts two types of stress in its graphical output: (1) randomly fluctuating (according to a z-distribution) week to week stress and (2) discrete elevations in stress intended to represent severe life events of the sort that might activate an adaptive depressive response. In animations accompanying this manuscript these are represented by visible elevations above a baseline stress level (both depicted in red) presented in the accompanying animations. Each “patch” in the NetLogo “world” has a variable that holds a numerical value for both types of stress, and a variable representing total stress (the sum of these two values). In NetLogo, the simulation space consists of “patches” that, together, comprise the “world” in which the simulation unfolds. The model depicts time on the x-axis of the “world” in order to allow the left to right movement of the agents to represent the mobile agents’ experiences over time. Each step conceptually represents one week and the entire world spans 520 weeks, or approximately 10 years. The threshold for activation (Nesse’s d-prime concept from the previous model) can be set to a specific value, or the agent can calibrate its response to the environment that it senses. More information about the model is contained in Additional file 3. Development of a function allowing agents to calibrate their responsiveness is described in more detail in Additional file 4. The dataset used in this calibration process is available as Additional file 5. The calibration function was used in animations presented later in the paper to compare agents capable of self-calibrating to those with immutable high or low thresholds.

As the mobile agents move through time, they activate their depressive response by setting their trajectory upward. The height of its elevation represents the severity of depression and plays a role in the way the disutility function is calculated. A well-adapted agent will track the pattern of stress very closely, as described in Additional file 3. A well-calibrated agent will rarely activate in the absence of a severe event and will promptly activate in the face of one.

## Results

Four video files are presented to illustrate output from the model. Each video depicts three types of mobile agent as they attempt to navigate their environment. These are screen captures of single agent simulations run in NetLogo. The model used to produce the simulations is available as Additional file 6. The top panel in each animation depicts an agent with a high threshold for activation. This agent has a threshold set to 6. As a value of 6 is unlikely to emerge from a z-distribution, these agents will have very few false positive activations. However, they tend to react slowly or fail to activate in the presence of a severe event. The bottom panel has a low threshold, set to 2.0, a non-extreme value from a z-distribution so the agents’ activation process is sensitive but not very specific. Finally, the middle panel depicts an agent that is able to sense its environment and adjust its threshold through a process of calibration. This agent starts with an intermediate threshold of four, counts the number of severe events, calculates a rate of their occurrence and sets its threshold at a value that is optimal for the rate it senses, according to a formula whose derivation as described in Additional file 4.

In an initial scenario, there are no severe events in the environment. In this case, the high threshold agents are most successful. The low threshold agents accumulate more disutility because of false positives. The calibrating agents do not calibrate in response to any events, so their baseline threshold value does not change during the simulation. They rarely activate since a value of 4 is unusual and so perform almost as well as the high threshold agents, see animation here [22], also available here [23].

In the next scenario there is a low frequency of events (*n* = 2). Again, agents with low thresholds experience many false positive activations. Agents with high thresholds and the self-calibrating agents are roughly equivalent in the accumulation of disutility, and both acquire less disutility than the low threshold agents. This is illustrated in a video available here [24], also available here [25]. A third scenario depicts a situation in which four severe events occur. The low threshold agent continues to accumulate excess disutility due to false positive activations, but now the high threshold agent is more strongly penalized for late activation or failing to activate in response to the events. The calibrating agents adapt to the environment, leading to a lower level of cumulative disutility than the immutably low or high threshold agents do. This scenario is depicted here [26], also available here [27]. A third scenario depicts 6 stressful events. Again, the agents that are able to calibrate their thresholds are able to minimize their disutility, in this scenario, the high-threshold agents perform particularly poorly but the adaptable ones perform similarly to the low threshold agents, see [28], also available here [29]. The ability to calibrate their responses allows the agents to adapt to a plurality of environmental conditions. 

However, the calibration process creates a vulnerability to problems. In the next scenario, a series of four events clusters in the first half of the simulation interval, after which they do not occur. Here, the adaptation process is counterproductive when viewed from the perspective of the second half of the simulation. Since the early-interval adversities result in establishment of a threshold that is a poor fit for subsequent adaptation, and the calibrating agent is subsequently subject to numerous false positive activations, as depicted here [30], and is also available here [31]. This scenario evinces the risk-inducing aspects of early life adversity.

## Discussion

The concepts associated with signal detection indicate that if depression is a defense, and that its application is governed by principles of signal detection theory, episodes of depression may occur as an outcome of the normal functioning of the defensive mechanism. The threshold-setting that is central to this concept provides a dimensional context for depression that is different from the traditional categorical versus dimensional debate (in which diagnostic categories are placed in opposition to symptom scales). Here, the dimensions involve thresholds of responsiveness and the frequency, timing and severity of stressful events. These concepts should deter the diagnosis of depressive disorders based on single episodes. When thresholds for activation are low (a presumed underlying pathophysiology for depression in this model), false positive activations are predicted to occur in a pattern that suggests a depressive disorder.

Before discussing the implications of the concepts and simulations presented here, it should be acknowledged that signal detection theory, including its application to the conceptualization of depression, has been criticized in the recent literature [32]. This criticism focuses on a simplification inherent in classical signal depression models - that optimal thresholds for action (termed “activation” in the current paper) are constant, which leads to a prediction that the best threshold for activation would have a monotonic pattern, as in the adaptation function used in these simulations (see the final figure in Additional file 4. Using predator-prey interactions as an example, Trimmer et al. pointed out that in an environment in which encounters with predators is highly likely, the frequent activation of defenses might be maladaptive because it could affect reproductive fitness by diminishing an organisms reserves. They also pointed out that autocorrelations would often exist: the current probability of danger, for example, may be strongly related to recent risks. In all of these scenarios more accurate models should depict the optimal threshold as state dependent, leading to a theory called state dependent detection theory or SDDT. However, the model presented here includes some elements of this theoretical orientation. First, the simulations were restricted to a range of stressful events in which a monotonic pattern was observed (see Additional file 4). How realistic this is in the real world is uncertain. A core concept of the agent-based model is that of an adaptable threshold – which encompasses the general idea of state dependent signal detection. Similarly, the concept of auto-correlation is partially addressed in the current model because while the agents’ depressive responses are activated, they remain so for a period of time, equivalent to the threshold being set to zero for a period of time. SDDT may provide an opportunity to refine the type of model presented here to reflect additional aspects of state-dependent responsivity. For example, as severely stressful life events may be more dangerous for young people than old, and the optimal threshold may therefore increase with age, potentially explaining the well-known decline in depressive disorder prevalence with age. The concept of “kindling” [33] is another example of a state-dependent change that may be represented by changing thresholds. Kindling may represent a diminishing threshold with recurrent episodes of depression, noting that recent studies have questioned whether kindling actually exists for mood disorders. What has been interpreted as kindling in the past may merely have been a statistical artifact [34].

Another limitation of the model presented here is its adoption of an “all or none” perspective on depression. Depression has a range of severity, so a more advanced model would be able to represent not only its unpredictable occurrence among people with depressive disorders, but also its well-known variability in terms of severity and persistence. Nesse [12] has described a signal detection approach for a continuously expressed defense.

The “smoke detector” concept provides an interesting perspective on some of some of the ambiguities and controversies that surround the depressive disorders. One of these ambiguities is that major depression often resembles reactions to loss (e.g. bereavement), so much so that the distinction was dropped from DSM-5, amid controversy [35]. The signal detection concept does not attempt to distinguish “normal” from “abnormal” episodes, but rather posits that the depressive syndrome is adaptive response, the regulation of which is subject to error. The model therefore does not focus on characteristics of specific episodes, but rather their pattern of occurrence over time in relation to stressors. The model provides an implicit solution to the “problem” of differentiating depressive responses to severe stress from more apparently pathological episodes that occur without severely stressful precipitants.

As noted above, the model is also dimensional, but not dimensional in the traditional sense of conceptualizing symptom severity on an ordinal (i.e. rating scale) rather than binary (i.e. diagnostic category) scale. Instead, it posits a probabilistically dimensional nature. A person with a higher threshold, as might be seen as a more resilient individual, suggesting that the depressive syndrome would not often occur in the absence of a severe event, but the probability is not zero. It posits that people without any particular vulnerability to depression would experience such episodes occasionally, most often in response to life events, but not always. One may speculate that because a large majority of the population is not highly susceptible to depression, their episodes might nevertheless contribute a sizable proportion of episodes in the population. This may draw into question the significance of single episodes, although currently in DSM-5 a single episode leads to assignment of a lifelong diagnosis. Typically, epidemiological studies report that approximately 50% of people with lifetime Major Depressive Disorders have had single episodes. Suspicions that epidemiological studies systematically over-estimate the prevalence of depressive disorders [36] are thereby supported by these arguments.

Another interesting implication is that the model may help to understand the “false positive” activation of the depressive syndrome, not as a defect, but as a component of its intended functioning. Some false positive activations are an expected outcome of a calibration process, even the ones that produce the best balance of sensitivity and specificity in a particular environment. These occurrences would be troublesome to the person affected, but may not represent a biological abnormality and this may help to explain the failure to identify clinically useful imaging strategies and biomarkers for depressive disorders.

Some of the most interesting implications of the model arise due to its probabilistic nature, which includes stochastic elements. In one simulation, a series of early (in the simulation) life events led to a calibration that resulted in numerous false positive activations later, a pattern reminiscent of the effect of adverse childhood events on subsequent mental health. In this example, a calibration process developed in one period was not the right fit for another period. This may represent a situation seen with child abuse and its well-known later association with depression [37]. Similar patterns occur with other forms of childhood adversity [38]. These features of the model may be an example of a bias-variance trade-off that is widely discussed in machine learning and statistical learning literature. The smoke detector model posits essentially that the body may seek to develop a predictive strategy to anticipate the occurrence of severe life events, through threshold setting. The model is developed based on experience (in the case of depression perhaps during childhood and adolescence) but its purpose would be to apply its predictions to later periods. The most sophisticated strategies for prediction are inevitably imperfect in terms of their ability to predict future events from past data. Sophisticated biological mechanisms attempting to calibrate responsiveness early in life might lead to a “over fit” version of this biological “predictive model” that does not perform well subsequently. Frankenhuis and del Guidice [39] described this concept in different terms, stating that a change in environmental conditions may make an adaptation maladaptive, but so may “sampling” errors in which the environmental data upon which the calibration is based is not representative conditions that will later be encountered. In general, however, a testable hypothesis arising from the model is that the number of childhood adversities should correlate with the frequency of occurrence of subsequent depressive episodes.

It is interesting to speculate about the implications of the model for treatment. Patients often report that antidepressant medications seem to reduce their emotional reactivity to life events, an effect that is not always welcomed [40, 41] but is theoretically consistent with the subjective experience of the resetting of a biological threshold for activation of a response. Elevating a threshold for activation of a depressive reaction, if this is an action of antidepressants, might foster resiliency in the face of stressors – although such resiliency could also be viewed as false negative responsivity according to the model. It would certainly lead to a lower frequency of “out of the blue” depressive episodes by reducing the false positive rate. A message for clinicians, if this model is valid, is that they should expect depression treatments to reduce the frequency of episodes, but not to eliminate them completely. Clinicians who believe that their treatments are correcting a biological defect (the clinician’s illusion) may change medications when there is a relapse, guided by the idea that the medication is not, or has stopped, working. However, it may be working, for example by reducing sensitivity to life events. This hypothesis could be evaluated in research including comparisons of treatment algorithms in real world settings. The hypothesis would be that when a patient has a sustained positive response to an antidepressant and then experiences a relapse while continuing to take it, persisting with the treatment would be a more effective strategy than switching to a different one.

Cognitive-behavioral therapies address the issue of distorted thoughts (including pessimistic ones about the future), thereby perhaps refining the accuracy of the “smoke-detector” mechanism. Mindfulness based approaches may address the same issue by their focus on living in the moment (thereby opposing any role that the “smoke-detector” may have as a predictive mechanism).

This same feature may help to explain the very modest effects of antidepressants in the clinical trial literature since DSM’s algorithmic approach may lead to misclassification of clinical status and since any effect of the medications on threshold setting would be expected to unfold over longer periods of time than the typical 6–8 week clinical trial. Antidepressant withdrawal effects [42] may be related to these same issues.

The well-known negative effect of stressors on prognosis in depression is also predicted by the model. In versions of the model in which agents have the ability to adapt to their surroundings, the agents start with a baseline threshold that is then adjusted depending on the environment that they encounter. This baseline threshold may reflect genetic predisposition to depression. In the simulations presented in this paper, a single value (4) was used, but a random variable such as a normal distribution may better reflect the well-known polygenetic diathesis.

A strength of simulation is its ability to represent stochastic effects, but such models can be misleading. Further development of these ideas in a more mathematically rigorous framework would be valuable.

## Conclusions

The signal detection model and associated smoke detector principle provide an addition to the list of conceptual frameworks for depressive disorders. Its ultimate contribution to an emerging theory of depressive disorders remains to be determined. Nevertheless, it offers an opportunity to address several conceptual problems that have plagued the literature in this area. It is worthy of further exploration, validation and analysis.

## Additional files

Additional file 1: Basic features of the signal detection concept, depicted in an Excel spreadsheet. This is the spreadsheet used to produce Figures 1-3. Values in the first three rows of the spreadsheet can be modified to explore scenarios different from those presented in in the Figures. To reproduce Fig. 1 in the graphic labeled “A classical signal detection paradigm”, enter the threshold (d-prime) = 2 in the first row of the spreadsheet. To reproduce the values in Fig. 2, make sure that the costs in row 2 & 3 are both set to one and then successively enter the values 1, 1.5 and 3 into the threshold (d-prime) cell in the first row. To reproduce Figure 3, set the cost of severe event to 10 and then enter 1, 2 or 3 in the d-prime field. (XLSX 56 kb)

Additional file 2: The agent based model in NetLogo format. This is a basic version of the NetLogo model used in the analysis to explore the effects of different thresholds in different situations. It is designed to generate a large datafile used to explore the effect of model parameters on disutility. A more elaborate version of the model (capable of simulating several agents with different characteristics) was used in generating the animations that accompany the manuscript. (NLOGO 28 kb)

Additional file 3: A pdf document providing a description of the NetLogo model. This pdf documents explains how the model works. Another description is available in the model itself (included as Additional file 2) in its “info” tab. (PDF 457 kb)

Additional file 4: An MS Word document providing a more detailed description of how the function used by agents to calibrate to their responses. The analysis used data (Additional file 5) derived from the NetLogo model (Additional file 2) to derive a linear function that allows an agent to select an appropriate threshold to guide their responses to their environment. These agents are depicted in the animations in the manuscript as the middle of the three panels presented in those animations. (PDF 381 kb)

Additional file 5: A comma delimited ASCII file generated by the NetLogo model. This data file is the output from the model included in with the manuscript as Additional file 2 for use in the analysis leading to a calibration process for the agents, as described in Additional file 4. (CSV 202 kb)

Additional file 6 The NetLogo model used to produce the animations presented in the manuscript. This file is in NetLogo format – parameters can be adjusted and new animations generated. (NLOGO 31 kb)

## Abbreviations

DSM-5: Diagnostic and statistical manual of mental disorders, 5th edition
SDDM: State-dependent detection theory
