# Supplementary material for: The “Clinician’s illusion” and the epidemiology, diagnosis and treatment of depressive disorders
Source: BMC Psychiatry. 2018 Dec 20;18:395. doi: 10.1186/s12888-018-1969-3 (PMC6302305; doi:10.1186/s12888-018-1969-3)
Supplement: Supplementary file 3 — A pdf document providing a description of the NetLogo model. This pdf documents explains how the model works. Another description is available in the model itself (included as Additional file 2) in its “info” tab. (PDF 457 kb) [file 12888_2018_1969_MOESM3_ESM.pdf]

Additional file 3: More details about the agent-based simulation

A simulation model offers the opportunity to present stochastic (randomly varying) features, which is an attractive feature for modeling a problem such as depression that often has unpredictable manifestations. NetLogo was used in the simulation work [1]. NetLogo is freely available software developed at the Massachusetts Institute of Technology, initially as a classroom tool. This simulation platform was selected since it is flexible, easy to use and does not depend on proprietary software. It has good pedagogical value owing to its roots as a classroom tool. Another feature of NetLogo is its ability to display the simulations in a visual animation, increasing accessibility of the simulation results. NetLogo has become widely used in simulation studies, especially agent-based simulation [1]. Agent-based models have the same goals as other types of models: they represent a real-world system as a means of explaining aspects of that system, or answering questions about it [2]. However, agent-based (or individual-based) modeling focuses on individual agents rather than variables that directly represent characteristics of the larger system [2]. Characteristics of a system emerge from the interaction of individual agents within the model.

The modeling interface in NetLogo includes a "world" which in this project was used to represent a pattern of stress in an environment over time. The world consists of agents called "patches" (small squares collectively comprising the simulated world) and another type of agent, mobile agents, which NetLogo refers to as "turtles" (the name reflects the software's original use as school classroom teaching tool). In the model presented here, mobile agents represent individual people. The following paragraphs describe the model, and visual portrayals of the model are presented in video files presented later in the paper.

The model depicts two types of stress in its world: (1) randomly fluctuating (according to a z-distribution) week to week stress and (2) discrete elevations in stress designed to represent severe life

events of the sort that might activate an adaptive depressive response. Each patch has a variable that holds a numerical value for both types of stress, and a variable representing total stress (the sum of these two values). The model depicts time on the x-axis of the "world" in order to allow the left to right movement of the agents to represent the mobile agents' experiences over time. Each step conceptually represents one week and the entire world spans 520 weeks, or approximately 10 years. The model depicts total stress in a column of patches using red coloring on the "world" with the level of total stress being represented as the height of the red patches on the y-axis of the "world".

As the mobile agents move through time, their challenge is to detect the severe life events and respond quickly to them. They do so by applying a threshold for activation of an all or none response representing activation of a depressive syndrome. If the level of stress on the patch that they occupy exceeds this threshold, they can react by adjusting their trajectory to match the level of stress associated with a severe life event. Their position on the y-axis conceptually represents their level of depression, higher on the y-axis means more depressed. Since the week-to-week variation follows a z-distribution, the agent might react when the stress level sensed is greater than 4 or 5, values that would be unlikely to arise from a z-distribution. Setting a high threshold in this way would reduce the likelihood of false positive activations, but such agents may be slowly responsive or non-responsive to the severe events.

The model depicts the stressful events as a triangle, with a height and slope of the sides set by the user on the interface. The shape is of course artificial, but reflects one of the problems of signal detection: the response being delayed as the severity of a stressful event mounts over time. An important aspect of the smoke detector concept is that the goal is to detect a problem at an earlier stage. The idea of higher levels of stress occurring over time challenges the agents with the problem of either waiting for clearer evidence or reacting quickly. Each agent is able to detect the shape of the simulated events. If their response is immediate, their movement will therefore track the level of stress closely. However,

this task is challenging since baseline stress produces noise in the environment. An increased level of stress could be due to an extreme value in the week-to-week fluctuation of stress, or it could be early evidence of an emerging severe life event. For reasons of simplicity, the model represents severe life events using a single set of values (a perpendicular height of the triangle and slope of its sides) entered by the user on the model's interface.

The mobile agents face the problem of signal detection. The earlier they can detect the severe life event, the more adaptive their response to it can be. However, they must seek to accomplish this in a noisy environment and there is a cost associated with each mistake. Each agent can sense the value of total stress on the patch that it occupies, but cannot sense the future. The agents decide whether to activate their all-or-nothing response based exclusively on this total stress value. If the total stress level exceeds a threshold, the response is activated (an episode of depression occurs). If the turtle has a low threshold for response, then it will be more sensitive, but less specific in detecting the severe life events. If it has a high threshold, then it will be less sensitive but more specific. **Figure S3.1** depicts a stressful event, along with a (yellow) mobile agent and selected patterns of response (dashed white lines).

Figure S3.1 Possible patterns of response to a discrete elevation in stress from a simulated life event

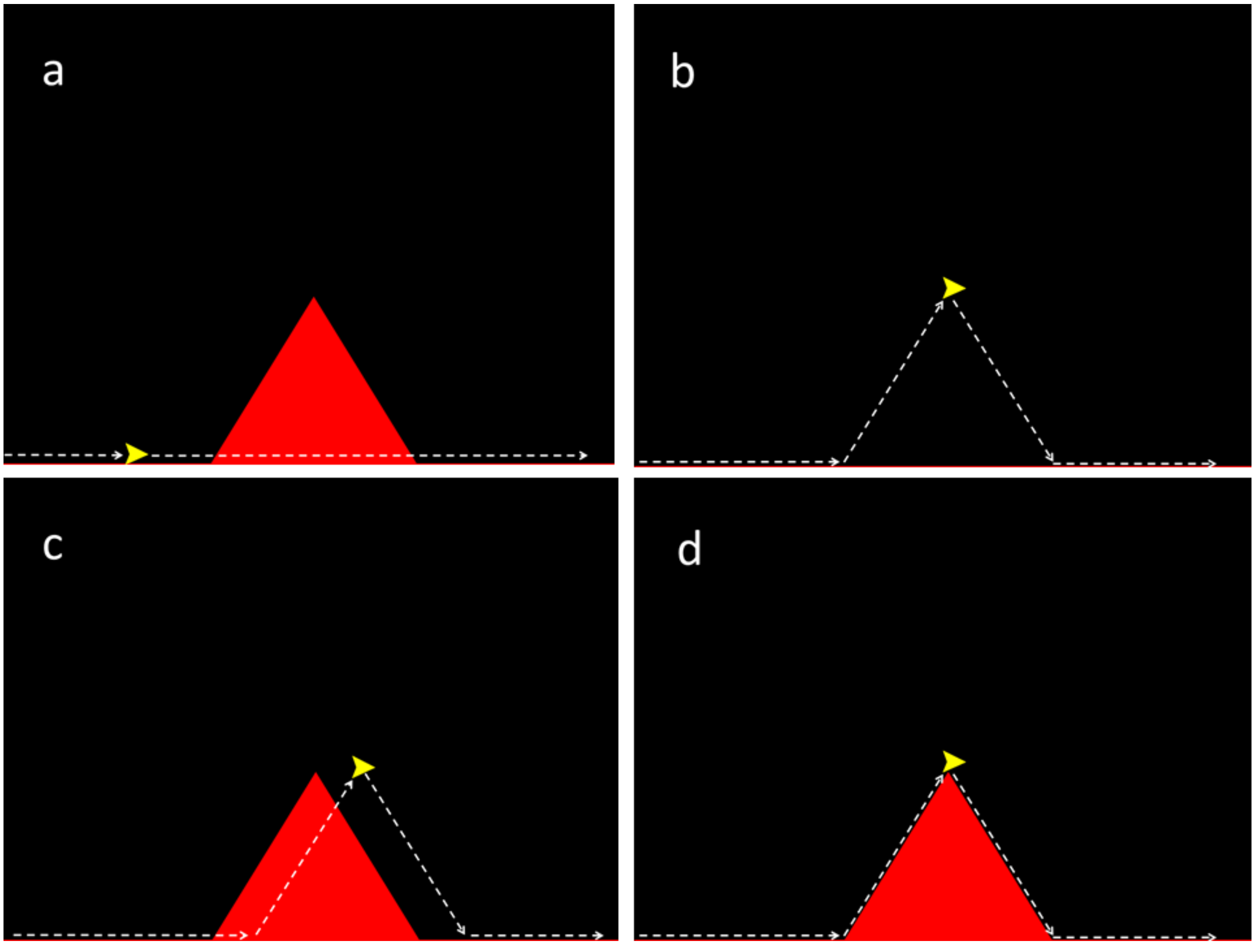

The model quantifies the success of its agents in navigating their environment using a disutility function. In the simulations, where level of depression is depicted by the height of the agent on the "world." Utility is maximized if the agent exactly follows the level of stress (see image 'd' from **Figure S3.1**). If their level of depression (recall that this is depicted by their height on the y-axis) is too low, then the extent of the difference at each time point is multiplied by a "too low" weight – representing the loss of utility due to not being depressed enough. On the other hand, the loss of utility associated with the depressed state is depicted at each time point as the level of depression multiplied by a weight representing the amount of disutility associated with depression (called depression-disutility), set on the model interface. These relationships are summarized in **Figure S3.2**. The extent of disutility is summed

at each of the 520 weekly steps of the model's simulation with the cumulative amount of disutility depicted graphically on the model's interface.

Figure S3.2 Calculation of disutility associated with optimal (a), absent (b), insufficient (c) or excessive (d) activation of a depressive syndrome*

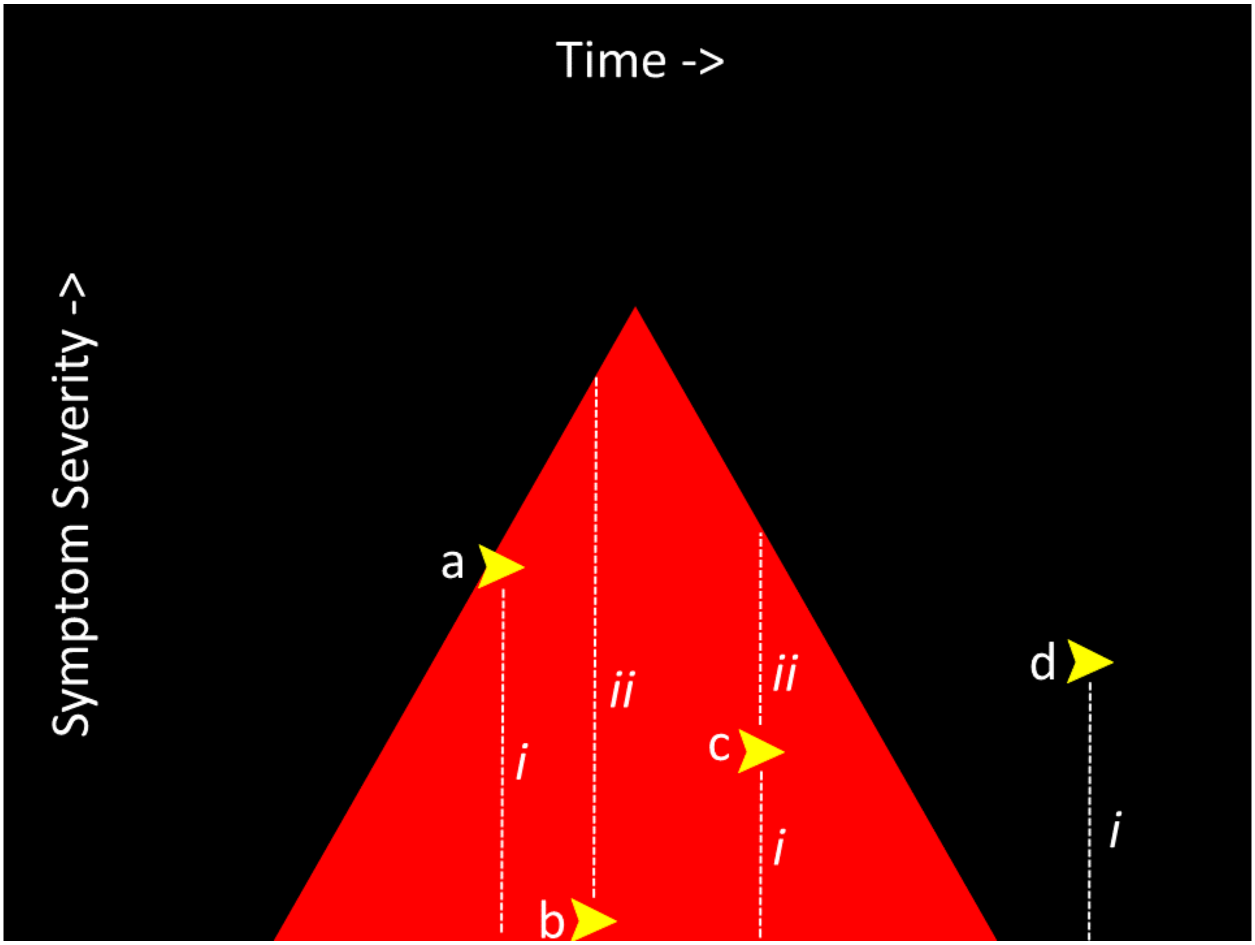

** A severe life event is depicted in red. The level of stress increases (left-hand side of figure), peaks (middle) and the resolves (right middle). The yellow shapes depict people as agents. The severity of the agents' depressive symptoms is represented by their vertical elevation in the figure. The white lines labelled *i* represent the loss of utility arising at a point in time from activation of the depressive syndrome whereas the white lines labelled *ii* represent loss of utility arising from inadequate activation of the depressive syndrome. Utility is lost due to a presumed adaptive purpose of depression. Agent (a) experiences the ill effects of depression, but has a level of depression that is optimally adaptive to the level of stress at this point in time. Agent (b) is not depressed and loses utility because the adaptive coping mechanisms is not invoked (false negative response). Agent (c) experiences both the loss of utility arising from depression but also a loss of utility for being insufficiently depressed to adapt optimally to the level of stress at this point in time. Agent (d) experiences a loss of utility because of depression, but as this agent is not experiencing a stressful event the depression has no adaptive function (false positive response).

Reference List

1. Wilensky U: **NetLogo**. In., 5.0.3 edn: The Center for Connected Learning (CCL) and Computer-based Modeling; 2012.
2. Railsback SF, Grimm G: **Models, agent-based models and the modeling cycle**. In: *Agent-based and individual-based modeling.* 1 edn. Woodstock: Princeton University Press; 2012.
