# Supplementary material for: The “Clinician’s illusion” and the epidemiology, diagnosis and treatment of depressive disorders
Source: BMC Psychiatry. 2018 Dec 20;18:395. doi: 10.1186/s12888-018-1969-3 (PMC6302305; doi:10.1186/s12888-018-1969-3)
Supplement: Supplementary file 4 — An MS Word document providing a more detailed description of how the function used by agents to calibrate to their responses. The analysis used data (Additional file 5) derived from the NetLogo model (Additional file 2) to derive a linear function that allows an agent to select an appropriate threshold to guide their responses to their environment. These agents are depicted in the animations in the manuscript as the middle of the three panels presented in those animations. (PDF 381 kb) [file 12888_2018_1969_MOESM4_ESM.pdf]

Additional file 4: A calibration process for the agents

Multiple simulations were conducted to explore the shape of the disutility function under various conditions. The model included with this manuscript as Additional file 2 includes the coding required to generate the dataset from which the functions presented below derive. The dataset is also attached as Additional file 5. When the rate of stressful events was zero, there was no minimum to the disutility function. This is shown in Figure S4.1. Since all activations are false positive activations, they all add disutility.

Figure S4.1. Zero events in environment

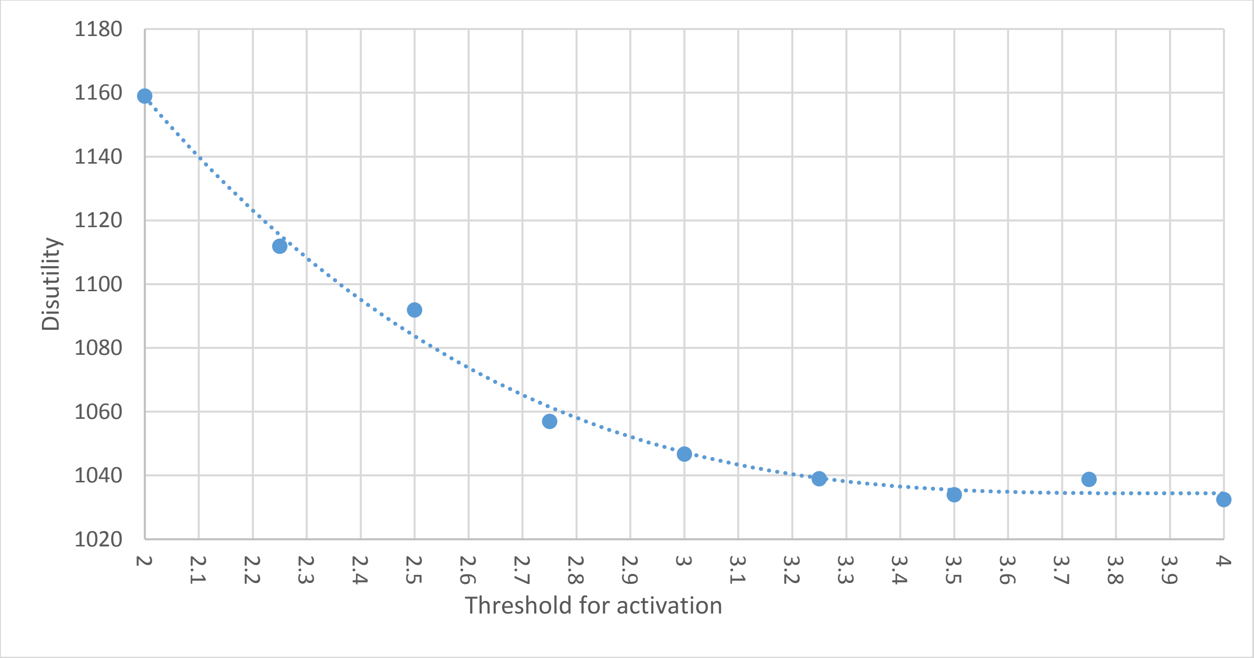

However, when the world contained and increased number of stressful events, disutility was maximized at ever-lower threshold. Figures S4.2, S4.3, S4.4, S4.5, S4.6 and S4.7 present disutility in worlds where there is 1, 2, 3, 4, 5 and 6 severe events, respectively. Each of these simulations is based upon 100 replications at a series of thresholds in environments with different numbers of events. Consistent with other simulation results reported in the paper, these simulations used the following parameters: the severe event height parameter was set to 5, the slope parameter to 0.5 (see model description, these parameters define the triangular distributions depicting the stressful events), the “too low” weight was

5, and the depression disutility weight was set to 1.0.The plots presented in this summary have a superimposed polynomial regression line. As the rate of events in the environment increases, the optimal thresholds that produce the minimum disutility get progressively smaller until at very high event rates, the optimal thresholds begin to approach two.

Figure S4.2 Disutility function in an environment with one severe event

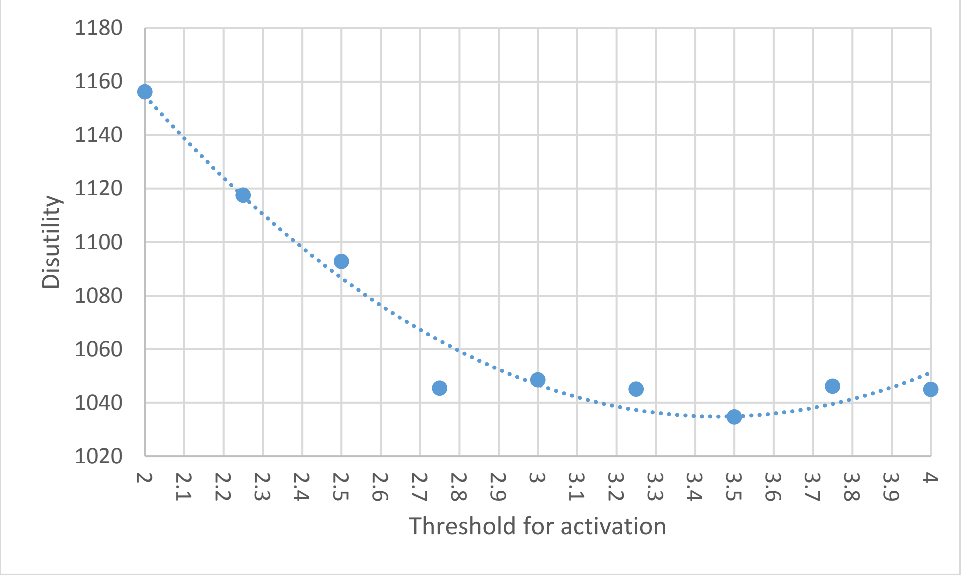

Figure S4.3 Disutility function in an environment with two severe events

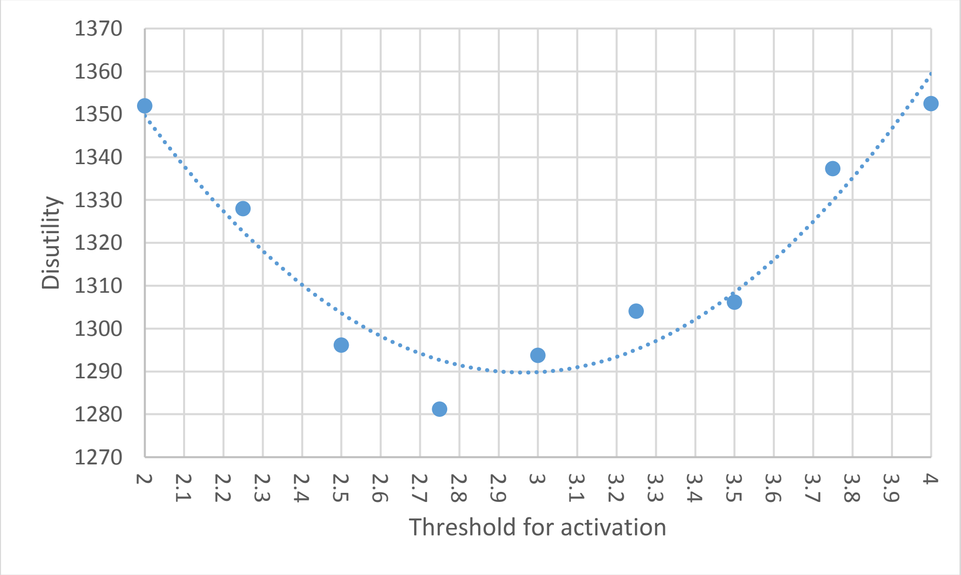

Figure S4.4 Disutility function in an environment with three severe events

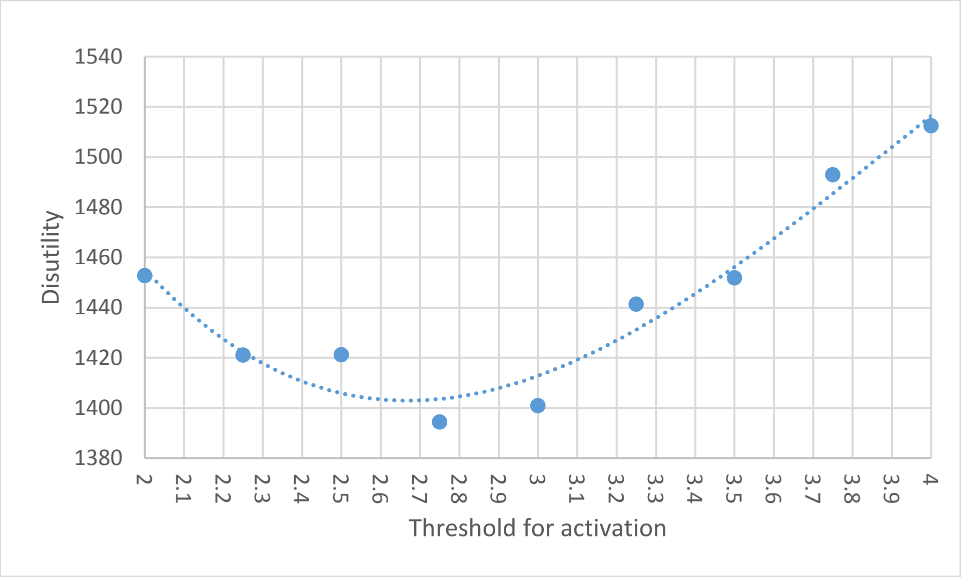

Figure S4.5 Disutility function in an environment with four severe events

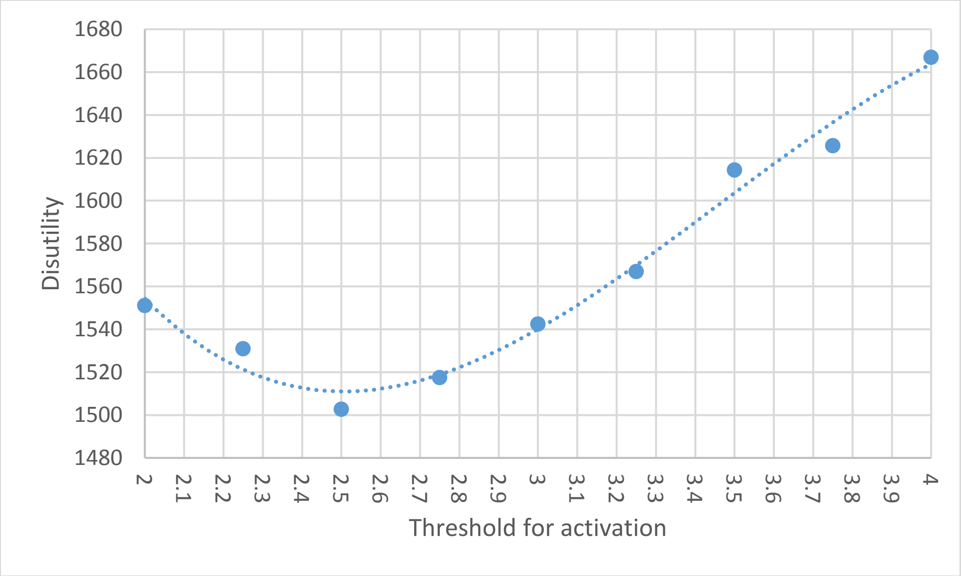

Figure S4.6 Disutility function in an environment with five severe events

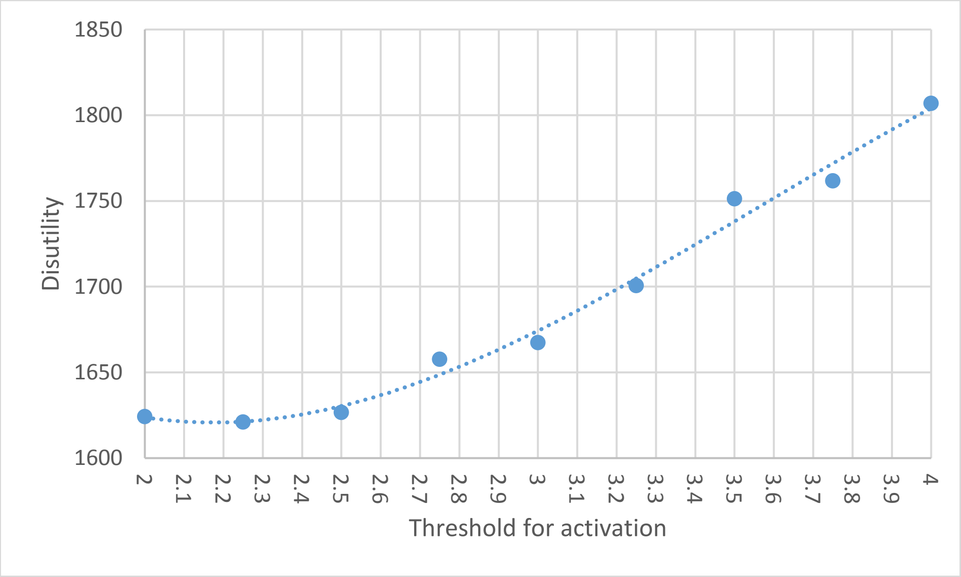

Figure S4.7 Disutility function in an environment with six severe events

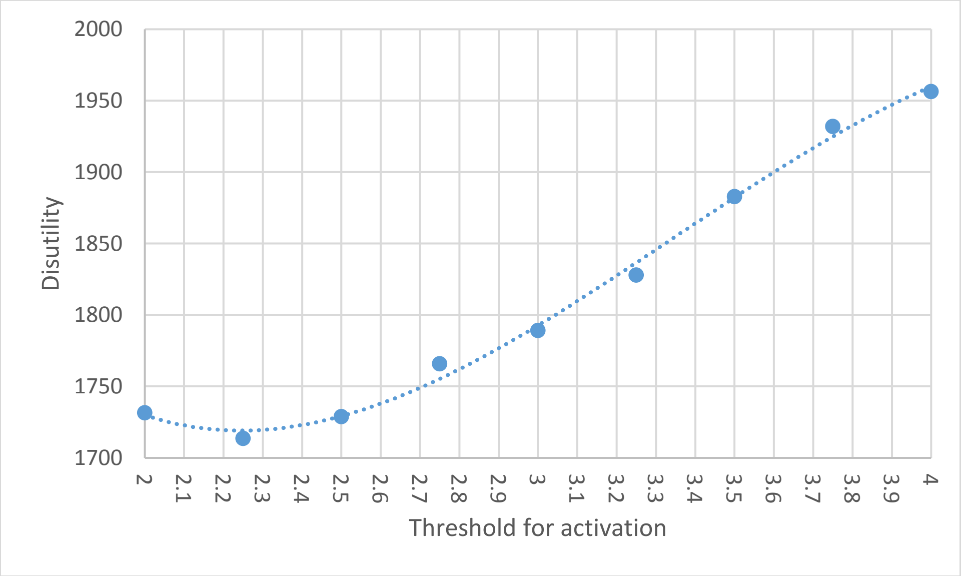

When the thresholds minimizing disutility at each event rate were plotted, an approximately linear relationship was seen, see **Figure S4.8**). This function is the basis of the adaptation process used by the adaptive agents. As they move through time, they estimate the event rate and alter their estimate each

time they encounter an event. By matching the event rates that they perceive in their environment to this linear function, the agents are able to calibrate their thresholds to the conditions in their environment.

Figure S4.8 An approximately linear relationship between the optimal threshold for activation and the event rate in the agents' environment

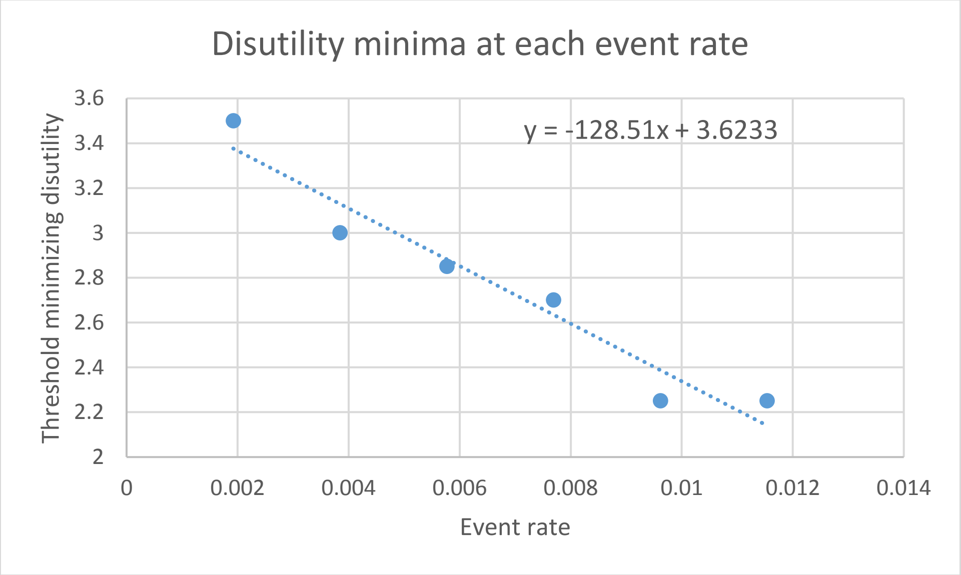

The version of the NetLogo model used to produce these results is attached as Additional file 2 and the data file (produced by this model and attached in comma delineated ASCII format) is Additional file 5.
